# The Quantitative Assessment of Imaging Features for the Study of Hirayama Disease Progression

**DOI:** 10.1155/2015/803148

**Published:** 2015-10-19

**Authors:** Minghao Shao, Jun Yin, Feizhou Lu, Chaojun Zheng, Hongli Wang, Jianyuan Jiang

**Affiliations:** Department of Orthopedics, Huashan Hospital, Fudan University, Shanghai 200040, China

## Abstract

*Objective.* To evaluate the forward shifting of cervical spinal cords in different segments of patients with Hirayama disease to determine whether the disease is self-limiting.* Methods.* This study was performed on 11 healthy subjects and 64 patients. According to the duration, the patients were divided into 5 groups (≤1 year, 1-2 years, 2-3 years, 3-4 years, and ≥4 years). Cervical magnetic resonance imaging (MRI) of flexion and conventional position was performed. The distances between the posterior edge of the spinal cord and the cervical spinal canal (*X*), the anterior and posterior wall of the cervical spinal canal (*Y*), and the anterior-posterior (*A*) and the transverse diameter (*B*) of spinal cord cross sections were measured at different cervical spinal segments (C4 to T1).* Results.* In cervical flexion position, a significant increase in *X*/*Y* of C4-5 segments was found in groups 2–5, the C5-6 and C6-7 segments in groups 1–5, and the C7-T1 segments in group 5 (*P* < 0.05). The degree of the increased *X*/*Y* and cervical flexion *X*/*Y* of C5-6 segments were different among the 5 groups (*P* < 0.05), which was likely due to rapid increases in *X*/*Y* during the course of Hirayama's disease.* Conclusion.* The *X*/*Y* change progression indicates that Hirayama disease may not be self-limiting.

## 1. Introduction

Hirayama disease (HD) is a juvenile muscular atrophy of the unilateral upper extremity first discovered by the Japanese scholar Hirayama in 1959; the disease was named after him [1].

Currently, the pathogenic mechanism of HD remains unclear. Hirayama et al. used autopsy to confirm that the major lesion of HD occurred primarily in locations such as the central white and gray matter and the ventral root of the anterior horn of the spinal cord [2, 3]. Combined with the special features of forward displacement of the spinal cord, an abnormal crescent signal posterior to the dural sac in flexion cervical magnetic resonance imaging (MRI) [3, 4], and features such as an age of disease onset 1–3 years after the peak of growth and development, they proposed that the unmatched growth rates of structures such as the spine, spinal cord, nerve root, and dural sac during the peak of growth and development resulted in the compression of the cervical spinal cord by the excessive forward displacement of the posterior wall of dural sac during cervical flexion movement. Additionally, the blood supply of corresponding segments of the spinal cord entered the crescent-shaped vacuum zone behind the spinal cord, causing compression and ischemia of the anterior horn of the cervical spinal cord, possibly indicating the major pathogenic mechanism of HD [3]. With the end of the growth and development peak, the relationship among the above anatomical structures gradually becomes balanced; therefore, the disease of HD patients gradually improves and becomes self-limiting after 2–4 years of disease onset [3, 5].

However, with the increase in HD cases and in-depth relevant studies, scholars have increasingly begun to question the “self-limitation” of HD. A large case study report confirmed that 7.5% of HD patients still had disease progression after a disease course of more than 5 years [5]. There are also a growing number of case reports concerning disease progression after a disease course of 10 or even 30 years [6, 7]. Some reports have even confirmed that patients with stationary disease could still experience rapid disease progression after several months or years of the plateau phase, eventually developing severe atrophy of the upper extremity [8, 9].

In 2006, in a large clinical case report involving Japan countrywide, Tashiro et al. accidentally found that, after disease progression (for several years in some HD patients), the degree of forward displacement of the posterior wall of the dural sac decreased during cervical flexion movement, thus proving that cervical spinal cord flexion might be a major pathogenic mechanism of HD and also showing that the “self-limitation” of HD might be attributed to the remission and disappearance of pathogenic factors. Thus, the special imaging features gradually became normal [10]. Unfortunately, Tashiro et al. did not perform further detailed studies on this issue.

Until now, there has been a lack of direct study evidence to confirm whether the imaging features that are closely associated with pathogenic factors of HD improve and become normal over time—that is, whether HD has “self-limitation.” Thus, in this study, we aimed to measure and compare the changes in the ratio between the anteroposterior diameter and transverse diameter of the cross section of spinal cord at the neutral position and flexion position of each cervical vertebral segment in HD patients with different disease courses and to measure the ratio between the distance from the posterior wall of the cervical spinal cord to the posterior wall of the vertebral canal and to the anterior and posterior walls of corresponding vertebral canal to quantitatively evaluate whether the above commonly seen abnormal structure of the cervical vertebral structure improved gradually with the progression of the disease course, thus elucidating whether the disease course of HD was “self-limiting.”

## 2. Patients and Methods

### 2.1. Patient Information

This study enrolled a total of 11 healthy volunteers (8 males and 3 females; age range, 21–35 years; body height range: 156–180 cm) and 64 HD patients (63 males and 1 female; age range, 16–26 years: body height range: 160–184 cm). Among the HD patients, 38 mainly had unilateral symptoms (average duration: 23.5 months), and the other 26 had significant muscular atrophy of the bilateral upper extremity (18 had more severe symptoms on the right side, and 8 had more severe symptoms on the left side). The disease course of the patients was 3–86 months (mean: 23.5 months), and 11 patients had disease courses ≥ 4 years. According to the disease course, patients were divided into 5 groups: ≤1 year (G1), 1-2 years (G2), 2-3 years (G3), 3-4 years (G4), and ≥4 (G5). All HD patients were treated at the Spinal Surgery Center of Huashan Hospital Affiliated with Fudan University between January 2011 and March 2014.

The inclusion criteria for HD were as follows: (1) the disease onset was earlier than 25 years of age; (2) the disease onset was insidious, and the disease course progressed a period of time (1 to 5 years); (3) patients had unbalanced limited weakness of the upper extremity or atrophy of the distal muscles and did not have combined sensory dysfunction; (4) patients could have combined clinical symptoms such as “cold paralysis” and “tremor on finger extension”; (5) neural changes were limited to unilateral or bilateral upper extremities, as suggested by neural electrophysiology, and primarily involved innervated sarcomeres at the C7-T1 segments and no abnormal detection of sensory nerves; (6) there was no motor neuron disease, cervical spondylosis, syringomyelia, spinal cord tumor, cervical deformity, polyneuropathy, focal neuropathy, brachial plexus injury, primary muscular dystrophy, trauma, inflammation, infection, or other disease history that caused the above clinical presentation.

### 2.2. Methods

**Figure 1:**
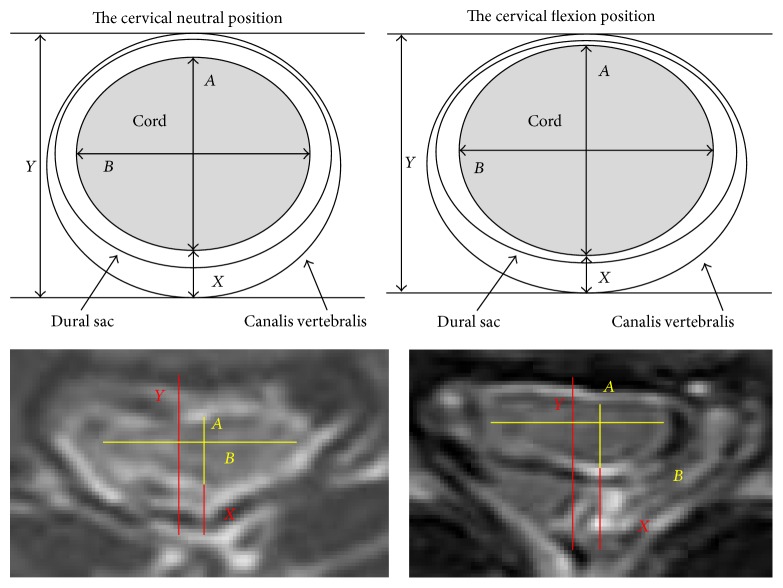
The distance between the posterior edge of the cervical spinal cord and posterior wall of the spinal canal was termed *X*, the distance between the anterior wall and posterior wall of the spinal canal was termed *Y*, and the anteroposterior diameter and transverse diameter of the cross section of spinal cord were termed *A* and *B*.

The study subjects assumed the supine position, and the muscles of the upper extremity were completely relaxed. Cervical MRI was performed under the cervical neutral position and flexion position (flexion for 45 degrees with the support of orthosis). The imaging parameters of the corresponding C4-5, C5-6, C6-7, and C7-T1 cervical vertebra segments were recorded and measured: the distance between the posterior edge of the cervical spinal cord and posterior wall of the spinal canal was termed *X*, the distance between the anterior wall and posterior wall of the spinal canal was termed *Y*, and the anteroposterior diameter and transverse diameter of the cross section of spinal cord were termed *A* and *B*, respectively (Figure 1). The cervical flexion position *X*/*Y* was used as the indicator for the observation of the range of abnormal signal images posterior to the spinal cord. The difference of *X*/*Y* between the cervical neutral position and flexion position was used as the indicator for the relative forward displacement degree of the cervical spinal cord. The flexion position *A*/*B* was used as the indicator for the observation of the relative morphological changes of the spinal cord. The number of HD patients who had a significantly increased abnormal signal posterior to the spinal cord is also counted.

All cervical vertebra MRI detection was performed using a 1.5 T Signa Excite MRI Machine (GE Healthcare, Milwaukee, WI, USA). The center of the magnetic field was set at the C6 level. The routine spin-echo sequence was used for scanning to obtain a transverse plane at T1-weighted and T2-weighted images. The scanning plane line was parallel to the connecting line of the intervertebral space. The T1-weighted parameters were TR/TE = 480 ms/9.2 ms, ST = 3.0 mm, and 512 *∗* 512. The T2-weighted parameters were TR/TE = 2700 ms/122.7 ms, ST = 3.0 mm, 512 *∗* 512, and FOV = 32 cm; the two-dimensional reconstruction was also completed.

### 2.3. Statistical Analyses

Statistical analyses were performed using the SPSS12.0 (SPSS, Chicago, IL, USA). The independent sample *t*-test was used for statistical analyses of the relevant parameters of each segment of the cervical vertebrae at the neutral position of the cervical vertebrae of healthy study subjects and HD patients with different disease courses. In addition, the paired *t*-test was used for the statistical analysis of each parameter item recorded at the neutral and flexion positions of the cervical vertebrae of HD patients in the different groups (or healthy study subjects). Statistical analyses of the relevant parameters among the different HD groups were performed using analysis of variance (ANOVA) and Kruskal-Wallis test. *P* < 0.05 was defined as indicating statistical significance.

## 3. Results

We performed MRI detection of cervical spine at the neutral position and flexion position in 11 healthy study subjects and 64 HD patients. The obtained data are listed in Table 1.

### 3.1. Healthy Study Subjects

In the healthy study subjects, none of the parameters recorded at the neutral position and flexion position of the cervical vertebrae have statistically significant differences (*P* > 0.05).

### 3.2. HD Patients

**Figure 2:**
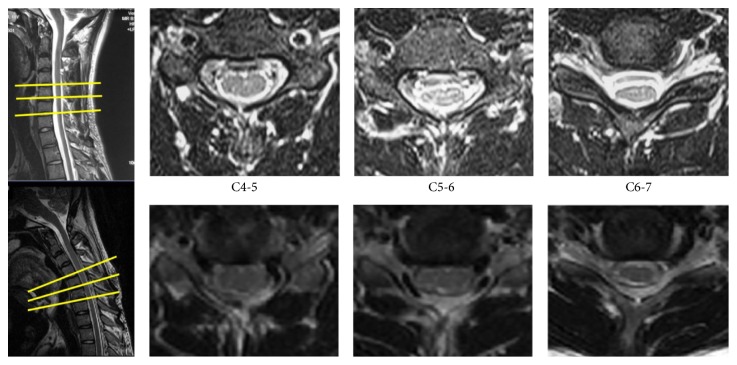
A case of 18-year-old man with Hirayama disease: T2-weighted sagittal and axial images showing a significant forward displacement of the spinal cord after cervical flexion movement and an increase in abnormal signal posterior to the dural sac.

**Figure 3:**
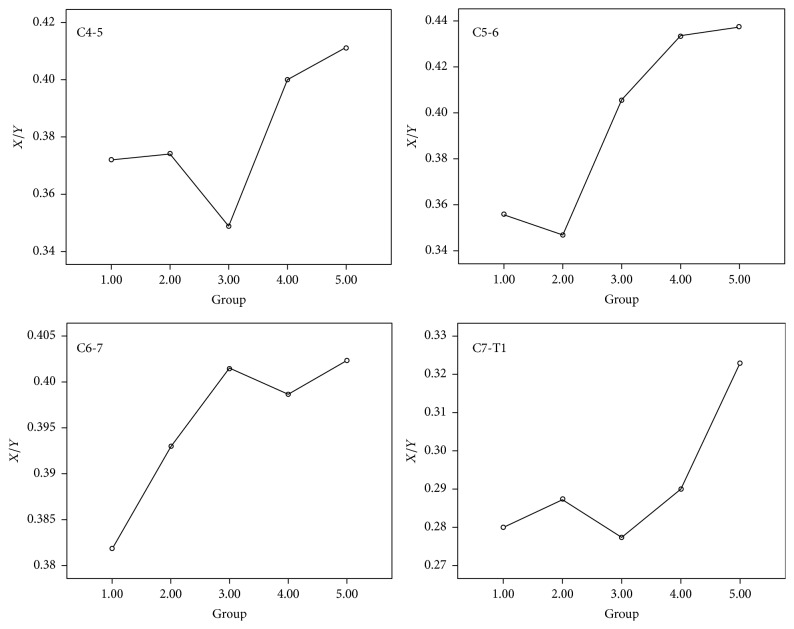
The mean *X*/*Y* (the parameters of the range forward displacement of the spinal cord) after cervical flexion movement.

**Figure 4:**
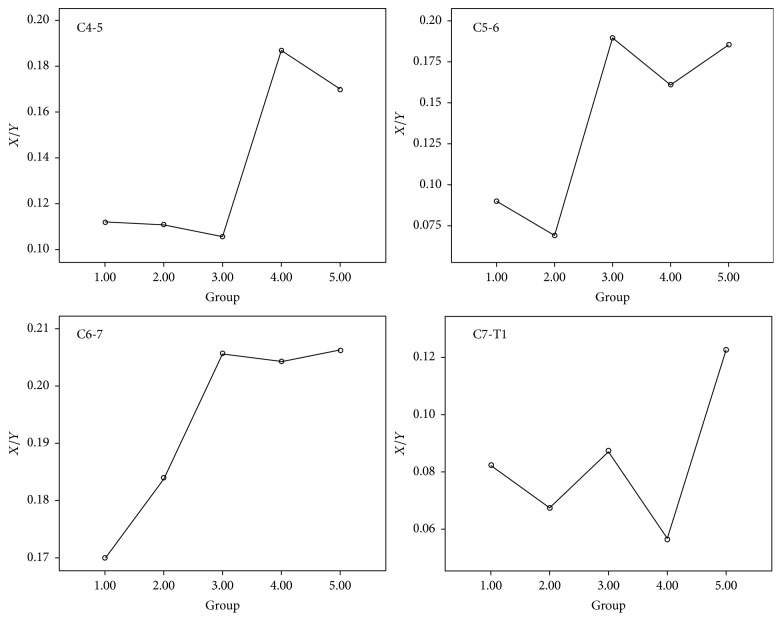
The mean difference of *X*/*Y* (the parameters of the range forward displacement of the spinal cord) between the cervical neutral position and cervical flexion position.

For the neutral position of the cervical vertebrae, the relevant parameters of the patients in each group did not show significant differences from those of the healthy population (*P* > 0.05). The ANOVA results showed that there was no significant difference between groups at the cervical vertebra neutral position (*P* > 0.05). For the C4-5 segment in the G2–G5 groups, the presentations of significant forward displacement of the cervical spinal cord and an increase in the abnormal signal posterior to the cord after cervical flexion movement occurred (*P* < 0.05); the abnormal imaging presentations identified above were significantly present in the C5-6 and C6-7 segments in the 5 groups after cervical flexion movement (*P* < 0.05) (Figure 2). The C7-T1 segment only presented with a significant forward displacement of the spinal cord after cervical flexion movement and an increase in abnormal signal image posterior to the spinal cord in patients with disease courses longer than 4 years (G5 group) (*P* < 0.05). The mean *X*/*Y* of C5-6 after cervical flexion movement among 5 groups has significant differences (*P* = 0.022). The mean difference of *X*/*Y* of C5-6 between the cervical neutral position and cervical flexion position among 5 groups also has significant differences (*P* = 0.030). It showed significant differences among these groups, and the mean plot showed that the above parameters were positively correlated with disease courses (*P* = 0.024) (Figures 3 and 4). At the flexion position of the cervical vertebrae, *X*/*Y* at the C4-5, C6-7, and C7-T1 segments among these groups did not show significant differences (*P* > 0.05); the differences in *X*/*Y* between the neutral position and flexion position of the cervical vertebrae at the C4-5, C6-7, and C7-T1 segments among these groups also did not show significant differences (*P* > 0.05).

The corresponding *A*/*B* of each segment of the cervical spinal cord of each group showed no significant difference (*P* > 0.05). In addition, *A*/*B* between all groups exhibited no significant difference (*P* > 0.05) (Table 2).

## 4. Discussion

In the present study, the parameters such as *X*/*Y* at the cervical flexion position and difference in *X*/*Y* between the neutral position and flexion position of the cervical vertebrae at the C4-T1 segments of each group did not decrease with the progression of the disease course. Therefore, the imaging features that were closely associated with the pathogenic factors of HD, such as the abnormal signal posterior to the dural sac and relative forward displacement of the cervical spinal cord in HD patients, might not improve along the disease course. Previous studies have shown that the abnormal signal posterior to the dural sac caused by the forward displacement of the cervical spinal cord results from the expansion of the venous plexus in the vacuum zone formed between the posterior wall of the dural sac and posterior wall of the spinal canal after cervical flexion movement [11, 12]. The resulting abnormal local hemodynamics in the cervical spinal cord are also currently the more recognized pathogenic mechanism [3]. In addition, this study showed that the obvious forward displacement of the cervical spinal cord after cervical flexion movement and abnormal signal posterior to the dural sac first occurs at the C5-6 and C6-7 segments (the corresponding cervical spinal cord segments are also the C7-T1 spinal cord segment that is most involved in HD, further confirming the close association between the above imaging features and pathogenic factors of HD). With the progression of the disease course, the C4-5 segment presents gradually, and the C7-T1 segment is clearly observed only when the disease course is longer than 4 years. This might explain the growing number of studies that have shown abnormal neural innervation at the deltoid muscle, sternocleidomastoid muscle, pectoralis major muscle, and thoracic paraspinal muscles at the advanced disease course of HD patients [11, 13]. The major explanation for the forward displacement of the cervical spinal cord after cervical flexion movement and continuous expansion of the venous plexus behind the dural sac might be the continuously repeated congestion and emptying of the venous plexus caused by repeated cervical flexion movement. During the cervical flexion movement, the pressure caused by the filling of the varicose veins would gradually separate the dura mater and spinal canal to form the abnormal forward displacement of the cervical spinal cord and abnormal signal posterior to the dural sac.

In the present study, only 10.9% (7/64) of HD patients had obvious morphological changes characterized by “flattening” of the cervical spinal cord after cervical flexion movement; that is, the *A*/*B* value was abnormal. In 2010, Lai et al. had already proposed that because the healthy population also had the presentation of forward displacement of the cervical spinal cord after cervical flexion movement, it was more reliable to diagnose HD through the observation of the morphological changes of “flattening” of the cervical spinal cord [13]. But their study only involved three HD patients. *A*/*B* reflects the ratio between the transverse diameter and anteroposterior diameter of the spinal cord. Some current studies have noted that some HD patients with longer disease courses might have thinning of the cervical spinal cord [6]; and morphological changes in the cervical spinal cord are also limited by factors such as the degree of compression and diameter of the cervical canal. That might explain why there was small amount of “flattening” observed in this present study. In addition, the “flattening” changes in the long-term disease courses could not appear due to thinning of the spinal cord. Therefore, the relative forward displacement of the cervical spinal cord and posterior “crescent” abnormal signal image at the flexion position under MRI may still be the most reliable indicators for the diagnosis and assessment of HD.

Flexion cervical MRI is currently the “gold standard” for the diagnosis and assessment of HD. A large case report has confirmed that, after cervical spinal cord flexion, the probability of the appearance of forward displacement of the spinal cord and an abnormal signal image posterior to the dural sac in HD patients is approximately 87–95% [5, 7]. Some scholars have even considered that because the pathogenic mechanism of HD remains unclear, only patients with the above specific image changes can be called “HD,” and others should be classified as the flexion type cervical spondylosis or unilateral muscular atrophy of the upper extremity [14]. Therefore, these authors have proposed that the probability of the presence of abnormal images in HD patients should be 100%. However, in the present study, this percentage was 71.9% (43/64).

Early studies noted that HD was “self-limiting” because the disease of HD patients gradually became stationary after 2–4 years. In addition, some scholars proposed that whether the disease course of HD was still at the progressive stage could be determined based on whether the detected muscles had spontaneous potentials, such as positive sharp waves and spike waves [9, 15]. Misra and Kalita even proposed that when the detected muscle no longer had spontaneous potential, it could be indirectly considered that the disease courses of HD had entered the stable stationary phase [15]; these proposals also provide the theoretical bases for the “self-limiting” of HD from the functional angle of the nerves. However, the disappearance of the spontaneous potential of detected muscles cannot show that the nerve damage has completely stopped because when the speed of muscle denervation is slower than that of innervation of the lateral buds, even with disease progression, the detected muscles would not have spontaneous potential. This condition is mostly observed in nerve root injury in chronic cervical and lumbar degenerative diseases. In this study, the mean plots showed that the C4-5, C5-6, and C-6-7 segments usually had the most significant forward displacement of the spinal cord. Although they still had persistent progression after more than 4 years, the slope between the G4 and G5 groups decreased more significantly than before, confirming that the progression slowed. However, the mean values clearly showed that when the separation of the 3 segments of the cervical vertebrae occurred to approximately 40% of the diameter of the spinal canal, the trend slowed. The study of Cerami et al. also considered that when the dura mater shifted forward to a certain degree, spinal cord atrophy occurred; subsequently, cervical spinal cord atrophy caused the disappearance of vascular compression, leading to the self-limitation of diseases [9]. Therefore, it could be considered that approximately 40% might be the limit of the forward displacement of the posterior wall of the dural sac. In addition, the mean plots showed that the C7-T1 segment showed a rapid increase in parameters such as *X*/*Y* after 4 years, reached an abnormal value from the original normal range, and exhibited rapid progression. Therefore, it could be considered that when the forward displacement of some segments of the cervical spinal cord or abnormal signal posterior to the dura mater reaches a certain limit, although the progression of this segment slows, subsequent spinal cord atrophy could occur, and the lesion range would not stop but expand to adjacent segments. That phenomenon could explain why, when many patients experience progression again at the plateau phase, the major presentations are atrophy in the forearm, shoulder, or thoracic or even thoracic paraspinal muscles. Therefore, for some cervical spinal cord segments, HD is “self-limiting”; however, the imaging presentation did not show remission with the progression of disease courses. Therefore, the remission of forward displacement cannot be regarded as evidence of “self-limitation.”

Application of the cross-sectional study was a significant limiting factor of this study. The presence of individual differences might significantly affect the experimental results. However, HD is a rare disease; when patients seek treatment, they usually already have severe muscular atrophy of the upper extremity or have already entered the plateau phase of the disease, making it difficult to perform longitudinal studies. In future studies, we will continue to collect additional cases of HD patients with confirmed diagnoses at the early stage of disease to further implement longitudinal studies with a large sample size.

This study confirmed that the forward displacement of the cervical spinal cord during flexion movement and increase in the posterior abnormal signal images were still the most sensitive and reliable indicators for the diagnosis and assessment of HD. However, with the progression of the disease courses, HD patients would not have a decrease of forward displacement of the cervical spinal cord. Although the expansion was somewhat limited for some segments, the forward displacement did not disappear with progression of the disease course; therefore, using the above reasons as the explanation for the “self-limiting” of HD is not sufficient. Although the degree of forward displacement is limited for each segment, the major pathogenic factors of HD may not disappear with the disease course, and some cases indicate that the condition might gradually expand to adjacent segments; thus, active treatment intervention measures should be performed. The major pathogenic factors of HD might not disappear with the disease course, but the condition might gradually become aggravated; thus, active treatment intervention measures should be performed.

## Figures and Tables

**Table 1:** The related parameters of Hirayama disease and healthy subjects in the cervical spine MRI.

	Hirayama disease										Normal subjects	
Course (year)	≤1		1-2		2-3		3-4		≥4		—	
*N*	10		20		14		9		11		11	
Age ± SD (range), yr	19.7 ± 2.7 (15–25)		18.3 ± 1.7 (15–21)		18.2 ± 1.5 (15–21)		19.9 ± 1.8 (18–22)		22.1 ± 2.5 (18–26)		36.5 ± 9.1 (21–52)	
Height ± SD (range), cm	172.3 ± 5.2 (160–183)		174.3 ± 5.1 (167–184)		172.7 ± 5.2 (162–182)		171.4 ± 3.5 (166–176)		173.2 ± 4.2 (168–180)		170.1 ± 6.6 (156–180)	
MRI	Neutral position	Flexion position	Neutral position	Flexion position	Neutral position	Flexion position	Neutral position	Flexion position	Neutral position	Flexion position	Neutral position	Flexion position

C4-5												
*A*/*B*	0.4 ± 0.1	0.5 ± 0.1	0.4 ± 0.1	0.5 ± 0.1	0.4 ± 0.1	0.4 ± 0.1	0.4 ± 0.1	0.4 ± 0.1	0.5 ± 0.1	0.5 ± 0.1	0.5 ± 0.1	0.5 ± 0.1
*X*/*Y*	0.3 ± 0.1	0.4 ± 0.1	0.3 ± 0.1	0.4 ± 0.1	0.2 ± 0.1	0.3 ± 0.1	0.2 ± 0.1	0.4 ± 0.1	0.2 ± 0.1	0.4 ± 0.1	0.2 ± 0.1	0.2 ± 0.1
C5-6												
*A*/*B*	0.4 ± 0.1	0.4 ± 0.1	0.4 ± 0.1	0.4 ± 0.1	0.4 ± 0.1	0.4 ± 0.1	0.4 ± 0.1	0.4 ± 0.1	0.4 ± 0.1	0.4 ± 0.1	0.4 ± 0.1	0.5 ± 0.1
*X*/*Y*	0.3 ± 0.1	0.4 ± 0.1	0.3 ± 0.04	0.3 ± 0.1	0.3 ± 0.1	0.4 ± 0.1	0.3 ± 0.1	0.4 ± 0.1	0.3 ± 0.1	0.4 ± 0.0	0.2 ± 0.1	0.2 ± 0.1
C6-7												
*A*/*B*	0.5 ± 0.1	0.5 ± 0.1	0.4 ± 0.1	0.4 ± 0.04	0.4 ± 0.1	0.5 ± 0.1	0.5 ± 0.1	0.5 ± 0.1	0.4 ± 0.1	0.4 ± 0.1	0.5 ± 0.1	0.5 ± 0.1
*X*/*Y*	0.2 ± 0.1	0.4 ± 0.1	0.2 ± 0.1	0.4 ± 0.1	0.2 ± 0.1	0.4 ± 0.1	0.2 ± 0.1	0.4 ± 0.1	0.2 ± 0.1	0.4 ± 0.0	0.2 ± 0.1	0.2 ± 0.1
C7-T1												
*A*/*B*	0.6 ± 0.1	0.6 ± 0.1	0.6 ± 0.1	0.6 ± 0.1	0.6 ± 0.1	0.6 ± 0.03	0.6 ± 0.1	0.6 ± 0.1	0.6 ± 0.1	0.5 ± 0.1	0.6 ± 0.1	0.6 ± 0.1
*X*/*Y*	0.2 ± 0.1	0.3 ± 0.1	0.2 ± 0.1	0.3 ± 0.1	0.2 ± 0.0	0.3 ± 0.1	0.2 ± 0.1	0.3 ± 0.1	0.2 ± 0.0	0.3 ± 0.1	0.2 ± 0.1	0.2 ± 0.1

Note: the distance between the posterior edge of the cervical spinal cord and posterior wall of the spinal canal was termed *X*, the distance between the anterior wall and posterior wall of the spinal canal was termed *Y*, and the anteroposterior diameter and transverse diameter of the cross section of spinal cord were termed *A* and *B*.

**Table 2:** At the cervical flexion position, the number of HD patients who had a significantly increased abnormal signal image posterior to the spinal cord is listed.

	C4-5			C5-6			C6-7			C7-T1		
	Neutral position	Flexion position	*P*	Neutral position	Flexion position	*P*	Neutral position	Flexion position	*P*	Neutral position	Flexion position	*P*
Course <1 year												
*A*/*B*	—	—	0.79	—	—	0.88	—	1	0.94	—	—	0.19
*X*/*Y*	—	3	0.15	—	4	0.04	—	6	0.03	—	2	0.32
Course 1-2 years												
*A*/*B*	—	—	0.21	—	—	0.47	1	1	0.81	—	—	0.27
*X*/*Y*	—	4	0.00	—	9	0.02	—	12	0.00	—	1	0.09
Course 2-3 years												
*A*/*B*	—	1	0.70	—	—	0.49	—	—	0.06	—	—	0.64
*X*/*Y*	—	6	0.03	—	8	0.01	—	6	0.00	—	1	0.22
Course 3-4 years												
*A*/*B*	—	—	0.10	1	2	0.19	—	1	0.60	—	—	0.91
*X*/*Y*	—	5	0.00	—	9	0.00	—	6	0.00	—	—	0.15
Course >4 years												
*A*/*B*	—	—	0.49	—	1	0.50	1	2	0.68	—	—	0.32
*X*/*Y*	—	7	0.00	—	11	0.00	—	9	0.00	—	5	0.02
